# Methylcobalamin promotes the differentiation of Schwann cells and remyelination in lysophosphatidylcholine-induced demyelination of the rat sciatic nerve

**DOI:** 10.3389/fncel.2015.00298

**Published:** 2015-08-04

**Authors:** Shunsuke Nishimoto, Hiroyuki Tanaka, Michio Okamoto, Kiyoshi Okada, Tsuyoshi Murase, Hideki Yoshikawa

**Affiliations:** ^1^Department of Orthopaedic Surgery, Graduate School of Medicine, Osaka University, SuitaJapan; ^2^Department of Orthopaedic Surgery, Toyonaka Municipal Hospital, ToyonakaJapan; ^3^Medical Center for Translational and Clinical Research, Osaka University Hospital, SuitaJapan

**Keywords:** methylcobalamin, peripheral nervous system, myelination, Erk signaling, cAMP, myelin basic protein, demyelinating disease

## Abstract

Schwann cells (SCs) are constituents of the peripheral nervous system. The differentiation of SCs in injured peripheral nerves is critical for regeneration after injury. Methylcobalamin (MeCbl) is a vitamin B12 analog that is necessary for the maintenance of the peripheral nervous system. In this study, we estimated the effect of MeCbl on SCs. We showed that MeCbl downregulated the activity of Erk1/2 and promoted the expression of the myelin basic protein in SCs. In a dorsal root ganglion neuron–SC coculture system, myelination was promoted by MeCbl. In a focal demyelination rat model, MeCbl promoted remyelination and motor and sensory functional regeneration. MeCbl promoted the *in vitro* differentiation of SCs and *in vivo* myelination in a rat demyelination model and may be a novel therapy for several types of nervous disorders.

## Introduction

Schwann cells are glial cells to form myelin in the peripheral nervous system. In SCs the differentiation process is precisely coordinated and differentiated SCs form a multispiraled extension of the plasma membrane to allow saltatory conduction (Jessen and Mirsky, 2005). Peripheral nerve injury may cause an axonal damage that may lead to Wallerian degeneration around the lesion site. Subsequently, it triggers a cascade including glial cell responses such as marked SC proliferation. SCs play an important role in the regeneration after peripheral nerve injury.

Vitamin B12 is crucial to maintain the normal function of the nervous system, and its deficiency leads to a systemic neuropathy called subacute combined degeneration of the spinal cord (Scalabrino et al., 1990). Moreover, vitamin B12 deficiency causes severe brain atrophy with symptoms of retarded myelination in a young child (Lovblad et al., 1997). MeCbl is an active form of vitamin B12 that is essential to the biochemical metabolism and prerequisite for motor and sensory functions of the mammalian nervous system (Weir and Scott, 1995; Izumi and Kaji, 2007). MeCbl is concerned with the reaction for the transmethylation, which converts from homocysteine to methionine, and has been shown to have a stronger affinity for nervous tissues compared with other analogs (Scalabrino and Peracchi, 2006). We previously reported that MeCbl is the most effective vitamin B12 analog for neurite outgrowth in cerebellar granule neurons and DRG neurons *in vitro* (Okada et al., 2010), with via the activation of Akt and the mammalian target of rapamycin (Okada et al., 2011). MeCbl also promoted nerve regeneration in *in vivo* nervous disorder models, such as streptozotocin-diabetic rats (Sonobe et al., 1988), acrylamide neuropathy rats (Watanabe et al., 1994), gracile axonal dystrophy mutant mice (Yamazaki et al., 1994), and sciatic nerve injured rats (Okada et al., 2010).

Furthermore, MeCbl can accelerate the myelination in the peripheral nervous system as it promotes the synthesis of lecithin, which is the chief ingredients of myelin sheath lipids, and enhances the peripheral nerve regeneration after injury in rats (Yamatsu et al., 1976; Watanabe et al., 1994; Reyes-Garcia et al., 2004). However, the precise mechanisms to promote MeCbl-mediated myelination are currently unknown.

In this study, we demonstrate a novel function of MeCbl, i.e., the promotion of the *in vitro* differentiation of SCs and remyelination in a LPC-induced local demyelination rat model. Our findings suggest that MeCbl promotes regeneration after peripheral nerve injury by promoting beneficial effects on not just neurons but also SCs.

## Materials and Methods

### Animals

Wistar rats (postnatal days 1–3, embryonic day 15 and 200 g adult; MF, Oriental Yeast, Osaka, Japan) were used. Animals were housed under a 12/12 h light/dark cycle (lights on, 08:00–20:00 h). All animals had free access to food (MF, Oriental Yeast, Osaka, Japan) and tap water. All experiments were performed in conformity to the guidelines of the Animal Care Committee of the Graduate School for Medicine, Osaka University. We made a maximum effort to minimize the number of animals used and to limit any suffering.

### Primary Culture of SCs

Primary rat SCs were isolated and cultured as previously described (Ogata et al., 2004). SCs were collected from the sciatic nerves of postnatal days 1–3 Wistar rats and cultured in Dulbecco’s Modified Eagle’s Medium (DMEM; GIBCO/BRL Life Technologies, Grand Island, NY, USA; not including vitamin B12) containing 10% fetal bovine serum (FBS; Sigma–Aldrich, St. Louis, MO, USA) and 1% penicillin and streptomycin. The following day, 10 μM cytosine arabinoside (Sigma–Aldrich) was added to the medium to eliminate contaminating fibroblasts. After 48 h, the medium was replaced with DMEM containing 3% FBS with 3 μM forskolin (Merck, Darmstadt, Germany) and 20 ng/mL of neuregulin (R&D Systems, Minneapolis, MN; growth medium) to expand the cells. Cells were then detached from the dishes using 0.25% trypsin (GIBCO/BRL Life Technologies) treatment and subculturing by replating at a 1:2 ratio onto poly-L-lysine-coated (Sigma–Aldrich) plastic dishes before confluence. We obtained a SC culture of >99% purity using these procedures. In all the experiments, cells were used between passages 3 and 8.

### Cell Proliferation Assay

Schwann cells were plated at a density of 1.4 × 10^4^ cells in 6-cm plates and maintained in the growth medium for 24 h prior to stimulation with MeCbl (100 μM; Sigma–Aldrich). On days 1, 3, and 5 after the stimulation, cells were trypsinized and resuspended. Cell counting was performed in triplicate on separate 10 μl aliquots using a hemacytometer.

### Western Blotting

Cultured SCs were collected and homogenized with 100-μL Kaplan buffer [150 mM NaCl, 50 mM Tris-HCl (pH 7.4), 1% NP-40, 10% glycerol, and a protease inhibitor cocktail] and clarified by centrifugation. Each sample included 18 μg of protein, was separated by SDS–PAGE, and transferred onto polyvinylidene difluoride membranes. After blocking non-specific binding sites with a blocking buffer [5% skimmed milk/1% Tween 20 in 20 mM TBS (pH 7.6)] for 1 h, the membranes were incubated overnight at 4°C with primary antibodies against p44/42 MAPK (1:1000; Cell Signaling Technology 4695, Beverly, MA, USA), phospho-p44/42 MAPK (1:1000; Cell Signaling Technology 9101), Akt (1:1000; Cell Signaling Technology 4691), phosphor-Akt (1:1000; Cell Signaling Technology 4056), GAPDH (1:1000; Cell Signaling Technology 2118), cleaved caspase-3 (Asp175; 1:1000; Cell Signaling Technology 9661), cleaved caspase-9 (Asp353; 1:1000; Cell Signaling Technology 9507), MAG (1:1000; Chemicon MAB1567), P0 (1:1000; Abcam ab31851, Cambridge, UK), MBP (1:1000; Sigma–Aldrich M3821) and Acly (1:1000; Abcam ab40793). Subsequently, the membranes were incubated with an anti-rabbit IgG, horseradish peroxidase linked whole antibody from donkey (1:1000; GE Healthcare Life Sciences NA934, Little Chalfont, UK) and subjected to ECL reagent treatment. Protein expression levels were determined using the MF-ChemiBIS 3.2 imaging system (Berthold Technologies, Bad Wildbad, Germany). The integrated optical densities of immunoreactive protein bands were measured using ImageJ 1.45 s, which is a public-domain image analysis program that was developed at the U.S. National Institutes of Health.

### Apoptosis Assay

Schwann cells were cultured on a poly-L-lysine-coated 35-mm plastic dish until they reached subconfluency. For the determination of apoptosis, cells were incubated with recombinant rat TNF-α, (100 ng/mL; Sigma–Aldrich) in the presence or absence of 100 μM MeCbl at 37°C for 24 h (Yuan et al., 2012). The effects of TNF-α on SCs apoptosis after treatment were determined by western blotting using an anti-cleaved caspase-3 antibody and anti-cleaved caspase-9 antibody.

### Differentiation Assay In *Vitro*

For differentiation experiments, purified cells were cultured in the growth medium for 24 h. They were induced to differentiate by the addition of db-cAMP (1 mM; Sigma–Aldrich; differentiation medium; (Yamauchi et al., 2011; Varela-Rey et al., 2014) and cultured for 72 h in the differentiation medium in the presence or absence of 100 μM MeCbl. The effects of the differentiation of SC after treatment were determined by western blotting using anti-MBP antibody and anti-Acly antibody.

### DRGs and SCs Coculture

Rat DRG neurons were prepared as previously described (Callizot et al., 2011), with some modifications. DRG neurons from the spinal cord of 15-day-old Wistar Rat embryos (E15) were sterilely extracted and treated with a trypsin solution (0.25% Trypsin-EDTA, GIBCO) at 37°C for 20 min; the reaction was stopped by the addition of DMEM containing DNase I grade 2 (0.1 mg/mL, Roche) and 10% FBS. Cells were mechanically dissociated by three forced passages through the tip of a 10-mL pipette. They were then centrifuged at 500 × *g* for 10 min. The supernatant was discarded and the pellet was resuspended in a defined culture medium containing neurobasal medium (Invitrogen, Carlsbad, CA, USA) supplemented with 2% B27 nutrient supplement (Invitrogen), L-glutamine (0.2 mmol/L; Invitrogen), 1% penicillin and streptomycin (10 mg/mL), and nerve growth factor (50 ng/mL; Millipore, Billerica, MA, USA). Viable cells were counted in an Invitrogen Countess^®^Automated Cell Counter. They were seeded at a density of 31,250 cells/well in an 8-well slide chamber that was precoated with poly-L-lysine and coated with laminin (1.4 g/cm^2^; Sigma–Aldrich) for 2 h at 37°C. Half of the medium was then changed every day. Cultures were maintained for 1 week to allow SCs to proliferate and unsheathe the axons of DRG neurons. At day 7, the cocultures were induced to form myelin via the addition of L-ascorbic acid (50 g/mL; Sigma–Aldrich) with or without MeCbl (100 μM), and half of the medium was then changed every day.

### Immunocytochemistry

Dorsal root ganglions/SCs cocultures on 8-well slide chambers were fixed with 4% paraformaldehyde for 10 min and permeabilized with 100% methanol for 30 min at -20°C. After blocking with PBS + 0.2% Triton X + 5% bovine serum albumin (Sigma–Aldrich), the samples were incubated with primary antibodies against MBP (1:1000; Calbiochem NE1018, San Diego, CA, USA) and NF200 (1:1000; Sigma–Aldrich N4142) overnight at 4°C, followed by incubation with the appropriate secondary antibodies including Alexa Fluor 488 goat anti-rabbit IgG (Molecular Probes A11034, Eugene, OR, USA) and Alexa Fluor 568 goat anti-mouse IgG (Molecular Probes A-11004). DAPI (Wako Pure Chemical Industries, Osaka, Japan) was included in the Permafluor (Thermo Fisher Scientific, Waltham, MA, USA) mounting solution to visualize nuclei. The number of MBP-positive segments was counted using the NIS Elements BR software (Laboratory Imaging, Nikon).

### Surgical Procedure

All animal experiments were approved by the Ethics Review Committee for Animal Experimentation of the Osaka University. Thirty-six male Wistar rats weighing 180–220 g were used in this study. For all experimental procedures, animals were deeply anesthetized using an intraperitoneal injection of a mixture of midazolam (2 mg/kg), butorphanol (2.5 mg/kg), and medetomidine (0.15 mg/kg). Under sterile conditions, the left sciatic nerve was exposed at the level of the sciatic notch. Using a Hamilton syringe, 5 μL of saline or 2% LPC (Sigma–Aldrich) in saline was injected into the proximal sciatic nerve. Seven days later, an osmotic minipump (Alzet, Cupertino, CA, USA) was placed subcutaneously in the back (Okada et al., 2010) to deliver continuous saline or MeCbl (1 mg/kg/day) for 1 week. All surgeries were performed by the same surgeon.

### Immunostaining of Sciatic Nerves

For histological evaluation of the lesions, animals were sacrificed 1 or 2 weeks after the LPC injection. They were deeply anesthetized, and the sciatic nerve containing the area of LPC application was excised for the evaluation of the extent of demyelination and remyelination. Sciatic nerves were fixed in 4% paraformaldehyde for 24 h at room temperature and then stored in 20% sucrose in 0.01 MPBS. The tissues were embedded in Tissue Tek (Sakura Finetek Japan), snap frozen on liquid nitrogen, sectioned axially at 5 μm, and mounted on a glass slide. They were permeabilized with 100% methanol for 30 min at -20°C. After blocking with PBS + 0.2% TritonX + 5% bovine serum albumin, they were incubated with primary antibodies against MBP (1:1000; Calbiochem NE1018) and NF200 (1:1000; Sigma–Aldrich N4142) overnight at 4°C inside a wet chamber, followed by incubation with the appropriate secondary antibodies including Alexa Fluor 488 goat anti-rabbit IgG, and Alexa Fluor 568 goat anti-mouse IgG. DAPI was included in the Permafluor mounting solution to visualize the nuclei. The number of myelinated axons was counted using the NIS Elements BR software.

### Sciatic Functional Index

Motor function was evaluated with the sciatic functional index as previously described (Varejao et al., 2001; Pereira Lopes et al., 2013; Goulart et al., 2014). Sciatic functional index was calculated at 2 weeks after the LPC injection. Rats were made to walk across a narrow track. The hind feet were dipped in block ink and changes in footprints were recorded on white papers. For normal footprints, a sciatic functional index value is near 0, whereas a sciatic functional index value of approximately -100 reflects complete loss of function (Bain et al., 1989).

### Electrophysiological Analysis

Under anesthesia, the sciatic nerve was exposed. A bipolar stimulating electrode was placed in the nerve trunk at its proximal portion and a recording electrode was placed in the anterior tibial muscle to record the compound muscle action potential. Nerve conduction velocity values were calculated by stimulating two different points of the sciatic nerve. Compound muscle action potential was detected and measured using the PowerLab devices and software (AD Instruments, Bella Vista, NSW, Australia).

### Behavioral Test

Responses to thermal stimuli were assessed using a hot plate (Ugo basile, Varese, Italy), at a temperature of 52.5°C. The reaction time (in seconds) until the first signs of a painful response (hindpaw licking or escape) was recorded and the cut-off time was 45 s (Guevara et al., 2015). Withdrawal thresholds of the hind paw to mechanical stimulation were determined using von Frey monofilaments (0.008–26 g; TouchTest, North Coast Medical Inc, Gilroy, CA, USA). Each filament was applied from beneath the mesh floor to the middle part of the plantar surface of each hind paw until the individual filaments used started to bend (Pitcher et al., 1999; Noda et al., 2014). Values are normalized to the unaffected side.

### Statistics

The JMP software, version 11 (SAS Institute, Cary, NC, USA) was used to analyze the results and the data were expressed as the mean ± SEM. The data were first analyzed for normality with Shapiro–Wilk test. Those qualified were then analyzed subsequently with one-way ANOVA followed by a *post hoc* Student’s *t*-test, Dunnett test, and Tukey–Kramer HSD test. Wilcoxon test and Steel test was only used when normality test failed.

## Results

### MeCbl does not Stimulate the Proliferation of SCs

**FIGURE 1:**
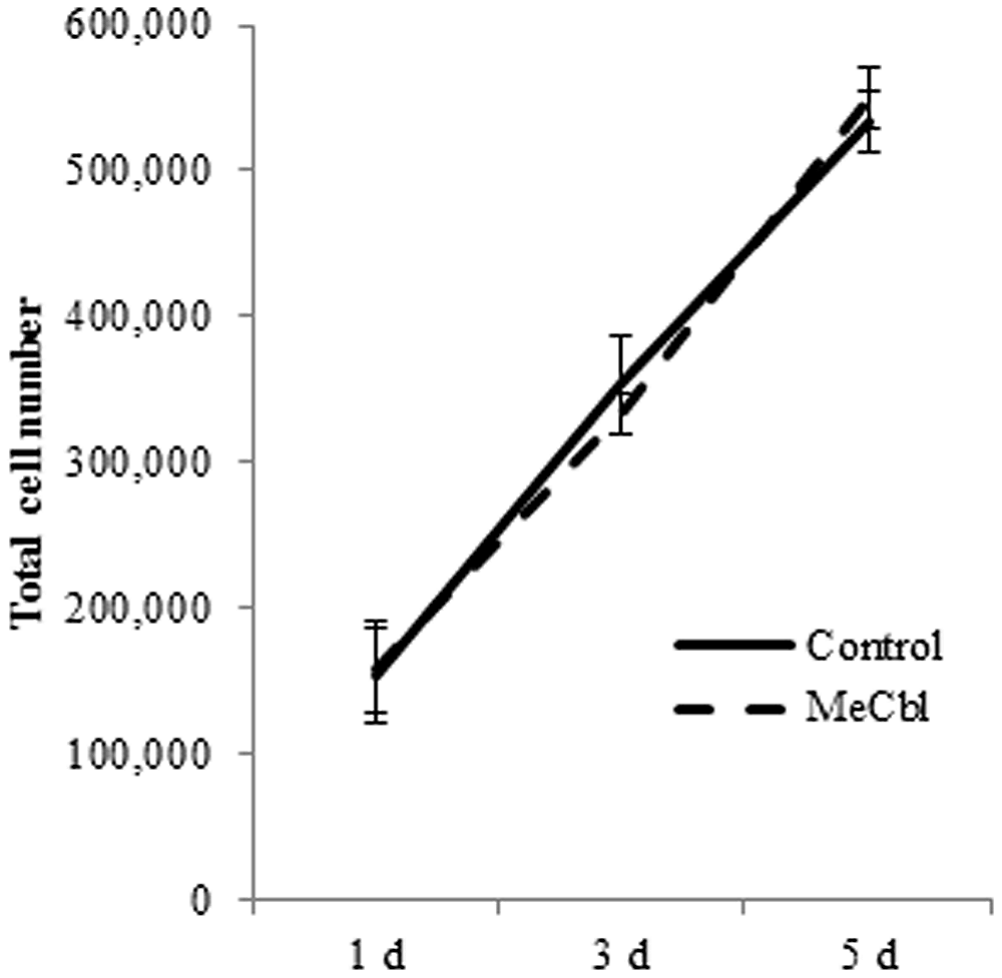
**Methylcobalamin does not stimulate the proliferation of SCs.** SCs were cultured in the growth medium for 5 days with or without 100 μM MeCbl and the total number of SCs was counted at days 1, 3, and 5. Values are means ± SEM of five independent experiments.

After peripheral nerve injury, SCs located in the distal nerve begin to dedifferentiate and proliferate; this reaction is a prerequisite process for the regeneration of damaged peripheral nerves. We focused on the effect of MeCbl on SC proliferation. SCs were cultured with or without 100 μM MeCbl for 5 days and the total cell number was then counted. The results indicate that there was no significant difference between the control and MeCbl groups on days 1, 3, and 5 (**Figure 1**). This finding suggests that MeCbl does not stimulate SC proliferation.

### MeCbl Reduces the Activity of Erk1/2 in SCs

**FIGURE 2:**
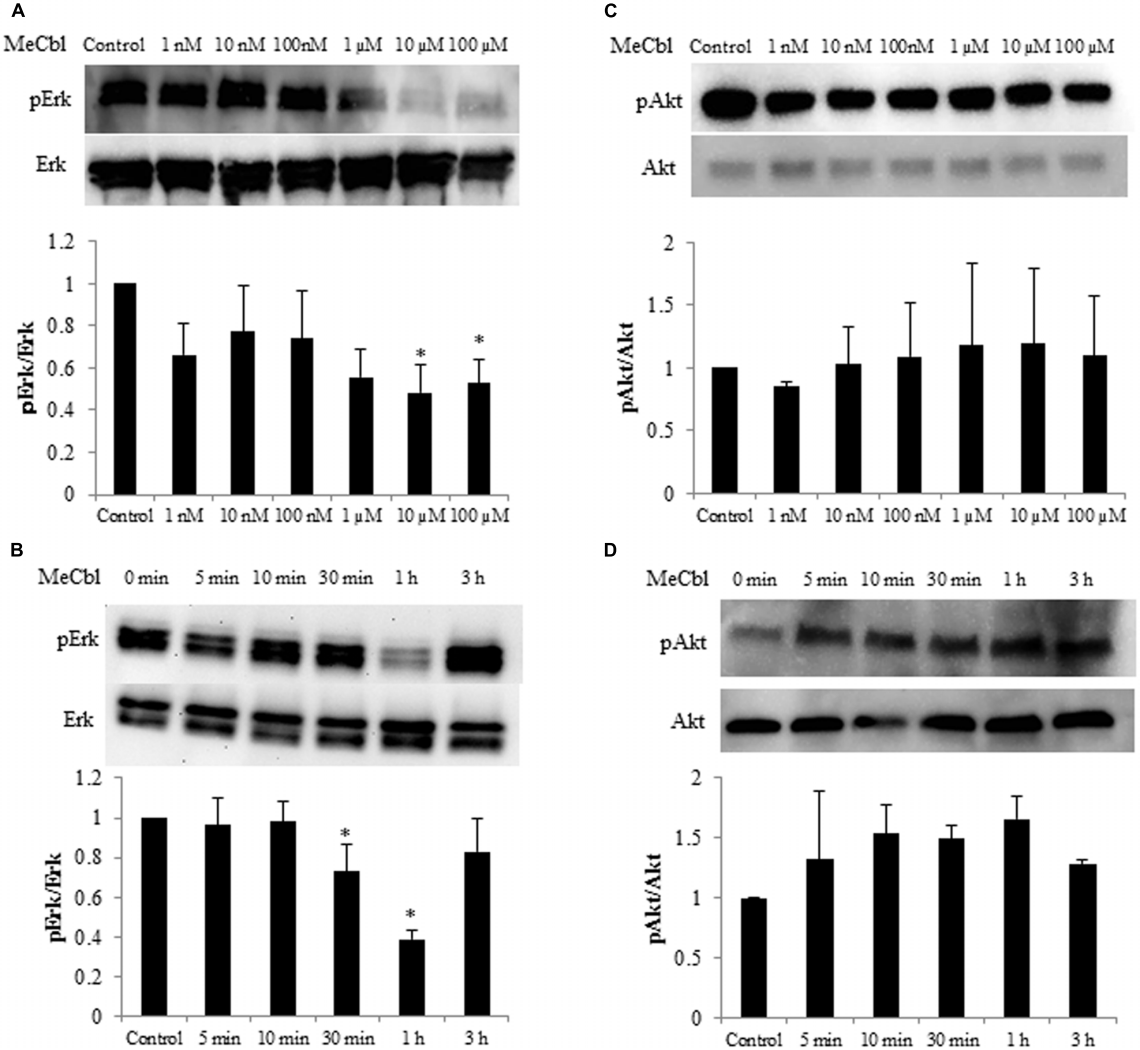
**Methylcobalamin reduces the activity of Erk1/2 in SCs. (A,C)** SCs were cultured with MeCbl at a concentration of 1 nM–100 μM for 1 h in the growth medium. Erk1/2 **(A)** and Akt **(C)** activities were detected by western blotting. The quantification of the normalized density of Erk1/2 **(A)** and Akt **(C)** is shown. **(B,D)** SCs were cultured with MeCbl at a concentration of 100 μM for 3 h in the growth medium. Erk1/2 **(B)** and Akt **(D)** activities were detected by western blotting. The quantification of the normalized density of Erk1/2 **(B)** and Akt **(D)** is shown. Values are means ± SEM of five independent experiments. *p < 0.05 compared with the control group.

Some studies identified the activation of the Erk1/2 pathway for SC proliferation (Ogata et al., 2004; Monje et al., 2006). To determine whether MeCbl influences Erk1/2 activity in SCs, we detected the activity of Erk1/2 in SCs cultured for 1 h with MeCbl at concentrations of 1 nM to 100 μM in the growth medium. We observed that MeCbl suppressed Erk1/2 activities in SCs at a concentration ≥10 μM (**Figure 2A**). Subsequently, SCs were cultured with 100 μM MeCbl for 3 h in the growth medium. MeCbl temporarily led to a 0.38 ± 0.04-fold weaker activation of Erk1/2 than that observed in the control at 1 h after the addition of the compound (**Figure 2B**). We next examined the activation of the Akt pathway which plays an important role in SCs differentiation (Ogata et al., 2004). We detected the activity of Akt in SCs cultured for 1 h with MeCbl at concentrations of 1 nM–100 μM (**Figure 2C**) and with 100 μM MeCbl for 3 h (**Figure 2D**) in the growth medium. The Akt activation was not detected in neither conditions (**Figures 2C,D**). These results clearly demonstrate that MeCbl suppresses the Erk1/2 activity in SCs, albeit temporarily.

### MeCbl does not Affect the TNF-α-Induced Apoptosis of SCs

**FIGURE 3:**
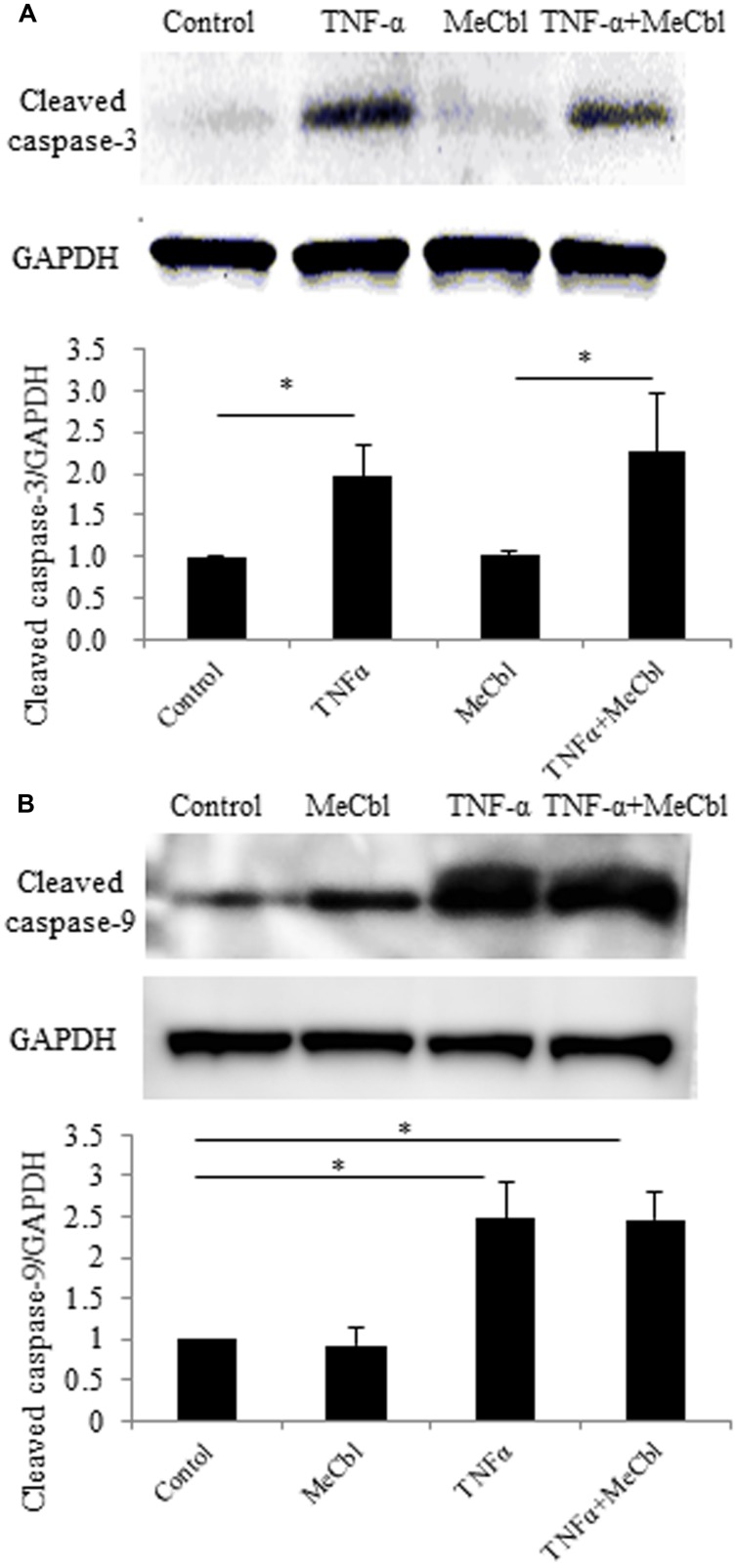
**Methylcobalamin does not affect the TNF-α-induced apoptosis of SCs.** SCs were stimulated with TNF-α for 24 h in the growth medium with or without 100 μM MeCbl. Cleaved caspase-3 **(A)** and cleaved caspase-9 **(B)** expression was detected by western blotting. The quantification of the normalized density of cleaved caspase-3 **(A)** and cleaved caspase-9 **(B)** is shown. Values are means ± SEM of six independent experiments. *p < 0.05.

The activation of Erk1/2 has been reported to protect against apoptosis in several cell types including neurons (Kolch, 2000; Xifro et al., 2014). To determine the effect of MeCbl on SC apoptosis, we cultured SCs with MeCbl (100 μM) in the presence or absence of TNF-α in the growth medium. We added TNF-α to the medium at a concentration of 100 ng/mL because TNF-α at a low concentration of 0.001 ng/mL induces SC proliferation, whereas TNF-α at a high concentration of 100 ng/mL induces SC apoptosis (Yuan et al., 2012). We examined the activation of cleaved caspase-3 and cleaved caspase-9, which were markers of apoptosis. MeCbl did not cause SC apoptosis, and TNF-α-induced apoptosis was not rescued by the administration of MeCbl (**Figures 3A,B**).

### MeCbl Upregulates the Expression of MBP and Acly in SCs under Differentiation Conditions *In Vitro*

**FIGURE 4:**
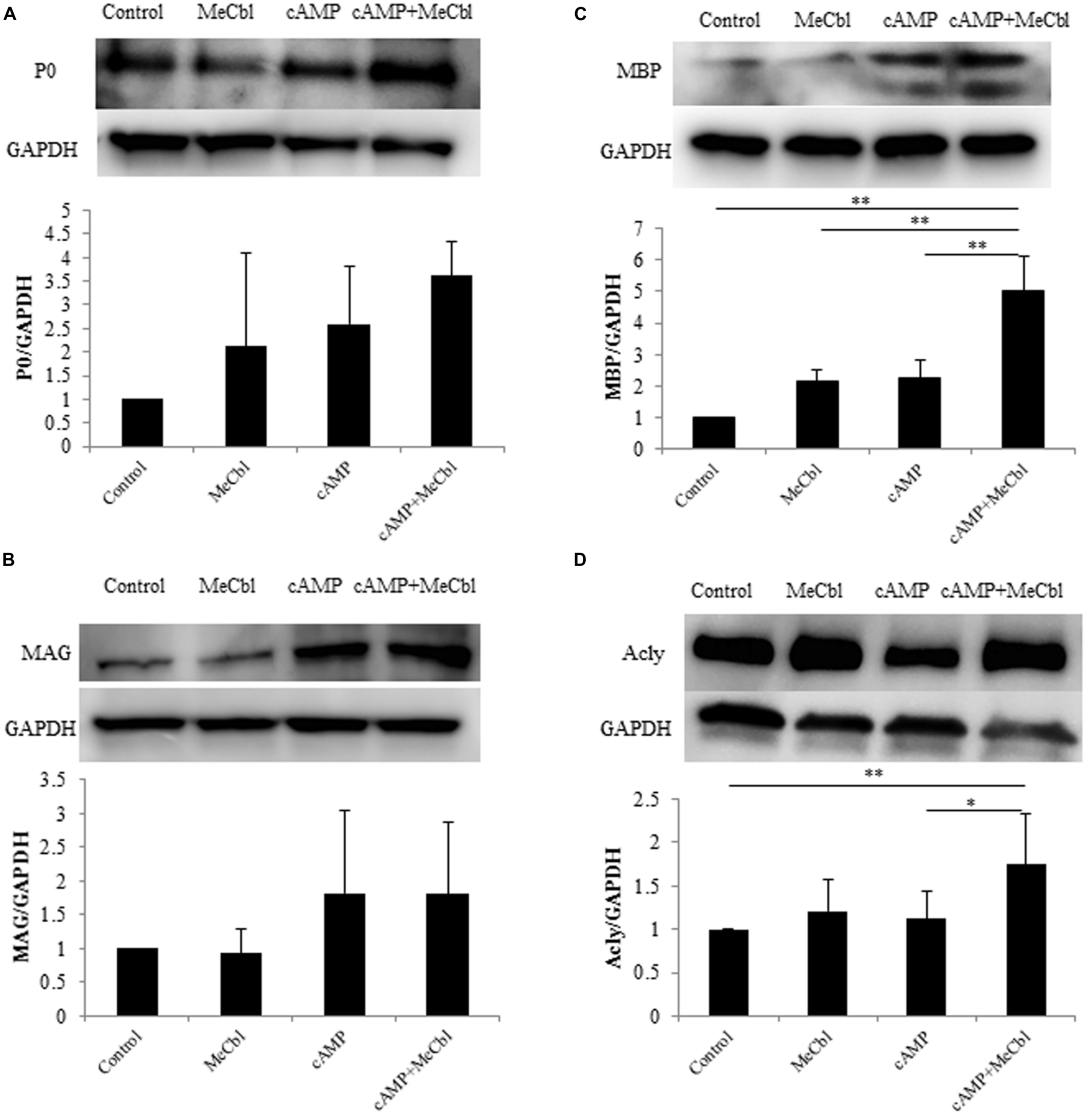
**Methylcobalamin upregulates the expression of MBP and Acly in SCs under differentiation conditions *in vitro*.** SCs were cultured in the growth medium for 24 h and in the differentiation medium including cAMP for 72 h with or without MeCbl (100 μM). The expression of P0 **(A)**, MAG **(B)**, MBP **(C)**, and Acly **(D)** was detected by western blotting. The quantification of the normalized density of P0 **(A)**, MAG **(B)**, MBP **(C)**, and Acly **(D)** is shown. Values are means ± SEM of more than three independent experiments. *p < 0.05, **p < 0.01.

Schwann cells are the main glial cells in the peripheral nervous system. After peripheral nerve injury, SCs first dedifferentiate and proliferate, and then redifferentiate and remyelinate newly grown axons in response to axon-derived signals, thus triggering a process of nerve regeneration (Harrisingh et al., 2004). cAMP is one of the signals that can mimic axonal contact with SCs and promotes the expression of the myelin marker galactocerebroside (Sobue and Pleasure, 1984) and a myelin protein (Ji et al., 2010; Yang et al., 2012) in SCs. First, we detected the expression of P0 and MAG, markers of SCs in the promyelinating state (Ogata et al., 2004). SCs were treated in the growth or the differentiation medium containing db-cAMP. MeCbl did not promote the expression of P0 (**Figure 4A**) and MAG (**Figure 4B**) in SCs in neither conditions. In SCs culture, P0 is clearly detectable even in the growth medium and the expression of MAG peaks at 48 h under the differentiation condition (Leitman et al., 2011). We therefore assumed that it might be difficult to detect the increasing expression of the markers in the promyelinating SCs by MeCbl and focused on the markers in the myelinating state. MBP is vital to the myelination process and is essential for the appropriate formation of myelin thickness and compactness in the central nervous system and peripheral nervous system (Zhang et al., 2007; Ryu et al., 2008). Therefore, we focused on the expression of MBP to evaluate the effect of MeCbl on SC differentiation. In the growth medium, MeCbl did not promote the expression of MBP in SCs (**Figure 4C**). On the other hand, the expression of MBP in SCs cultured in the differentiation medium was increased by the administration of MeCbl (**Figure 4C**).

Abundant lipid and cholesterol biosynthesis is necessary for the SCs myelination of peripheral nerves. Recent experiments using microarray analyses of the myelination process during development and the remyelination process after nerve injury have revealed that cholesterol/lipid metabolism in peripheral nerve myelination is also important (Nagarajan et al., 2002; Verheijen et al., 2003). Next, we examined the activation of Acly, which is a marker of lipid-synthesis enzymes. Together with the expression of MBP, MeCbl promoted the expression of Acly in SCs in the differentiation medium but not in the growth medium (**Figure 4D**). In addition, to clarify the increasing expression of MBP by MeCbl, we applied the following experiment.

### MeCbl Accelerates the Myelination of Cocultured DRGs/SCs

**FIGURE 5:**
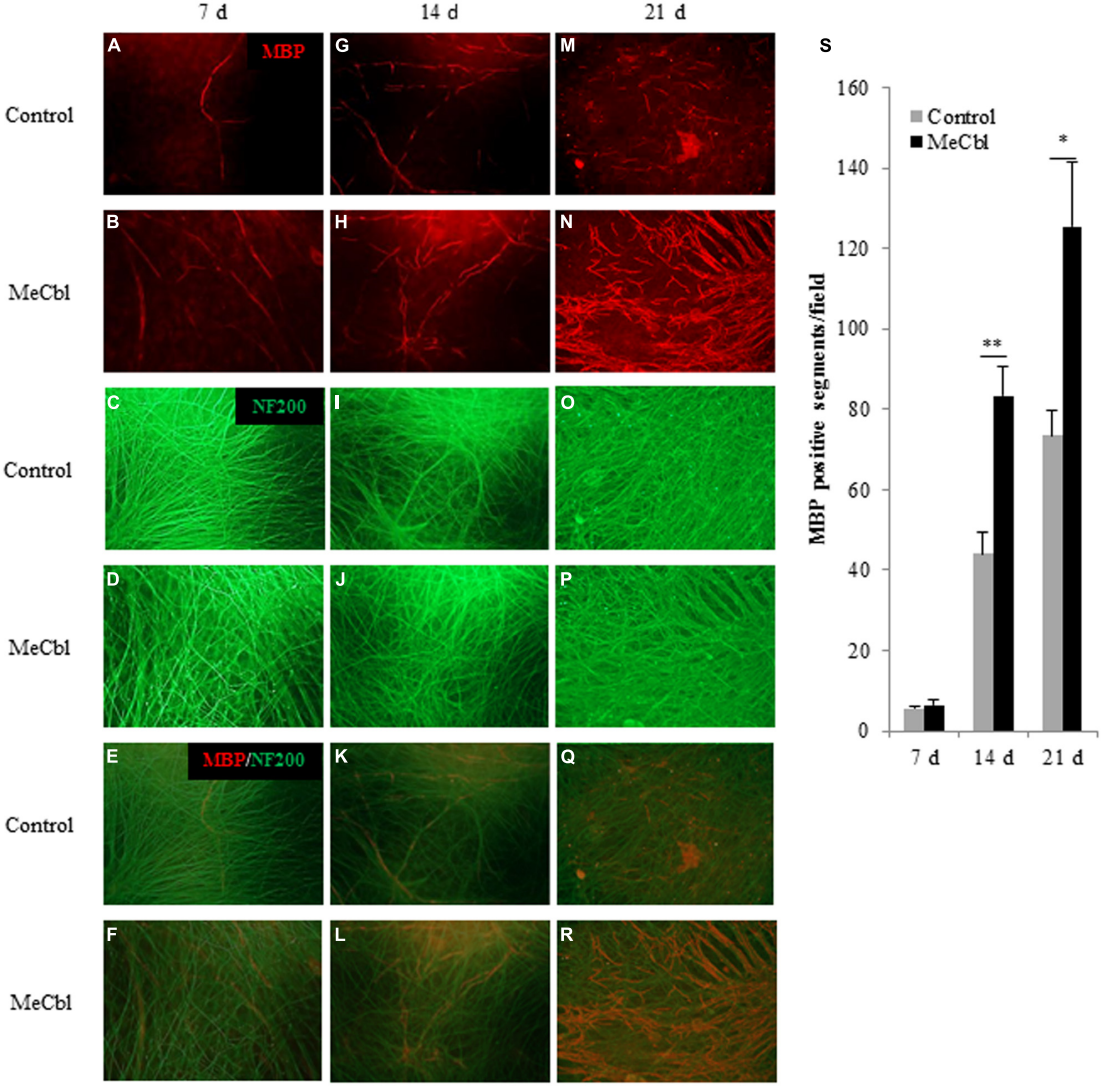
**Methylcobalamin accelerates the myelination of DRGs/SCs in coculture.** We used cocultures of DRGs and SCs to evaluate the differentiation of axon-related SCs in the presence or absence of 100 μM MeCbl. Cocultures were visualized with an anti-MBP antibody red **(A,B,G,H,M,N)** and an anti-NF200 antibody green **(C,D,I,J,O,P)** and merged pictures **(E,F,K,L,Q,R)** were shown at 7 **(A–F)**, 14 **(G–L)**, and 21 days **(M–R)** days after the induction of differentiation. **(S)** The extent of myelination was quantified by measuring the number of MBP-positive segments. Values are means ± SEM of five independent experiments. *p < 0.05, **p < 0.01 compared with the control group.

Early studies have shown that after the proliferation stage SCs differentiate into myelination process and its initiation is the stimulation by direct physical contact with axonal membranes (Wood and Bunge, 1975; Salzer et al., 1980). To date, the culture consisting SCs and primary DRG neurons is the unique and stable *in vitro* system and it enables to support both active proliferation and myelination by purified isolated populations of postnatal rodent SCs (Eldridge et al., 1987). Therefore, we used a DRGs/SCs coculture system to estimate MeCbl-mediated myelination. DRGs/SCs cocultures were maintained for 21 days to examine the effect of MeCbl on the differentiation of axon-related SCs. The extent of myelination was quantified by measuring the number of myelinated sections. No significant differences in the number of myelin segments were observed on day 7 (**Figures 5A–F,S**). On day 14 after the induction of differentiation, MeCbl increased the number of MBP-positive segments by approximately twofold compared with that observed by the control (**Figures 5G–L,S**). In both groups, the differentiation of SC progressed for 21 days and the number of MBP-positive segments in the MeCbl group was approximately 1.5-fold compared with that observed in the control group (**Figures 5M–S**). Collectively, these results provide strong evidence that MeCbl promotes the differentiation and myelination of SCs *in vitro*.

### MeCbl Promotes Regeneration after LPC-Induced Sciatic Nerve Demyelination *In Vivo*

**FIGURE 6:**
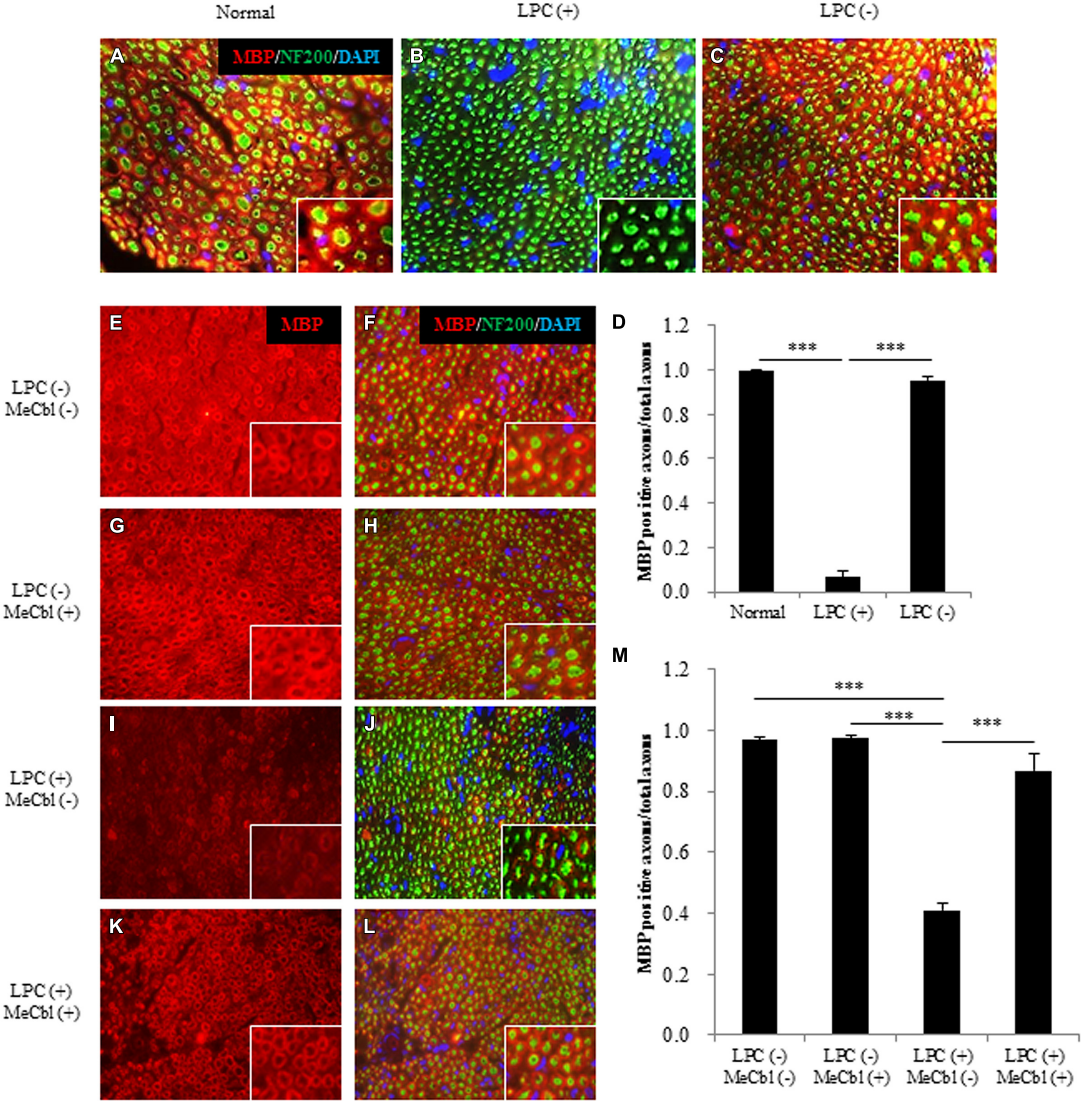
**Methylcobalamin promotes remyelination after LPC-induced sciatic nerve demyelination *in vivo*. (A–C)** Fluorescence micrographs of cross-sectional slices of sciatic nerves labeled for MBP (red), NF200 (green), and DAPI (blue) 7 days after the LPC [b; LPC (+)] or saline [c; LPC (-)] injection. Images taken at a higher magnification are shown in the insets. **(D)** Quantification of MBP-positive axons per total axons (NF200-positive axons) 7 days after the LPC or saline injection. **(E–L)** MeCbl or saline was administered systemically 7 days after the injection. Fluorescence micrographs of cross-sectional slices of sciatic nerves labeled for MBP red **(E–L)**, NF200 green **(F,H,J,L)**, and DAPI blue **(F,H,J,L)** 7 days after the administration of MeCbl or saline. Images taken at a higher magnification are shown in the insets. **(M)** Quantification of MBP-positive axons per total axons (NF200-positive axons) 7 days after the administration of MeCbl or saline. ***p < 0.001. Values are means ± SEM of three independent experiments.

To make the focal demyelination model of the sciatic nerve, we used LPC that dissolves myelin sheaths and leads to pure demyelination lesions which spontaneously remyelinate over time (Smith and Hall, 1980; Stoll et al., 1993; Wessig et al., 2007; Zhang et al., 2010). To confirm the effect of LPC, cross-sections of sciatic nerves were prepared and demyelination was assessed by immunofluorescence for MBP (**Figures 6A–D**). On 7 days postoperatively, MBP positive axons were clearly visible in saline injected LPC (-) group (**Figures 6C,D**), whereas it was rarely observed in the LPC (+) group (**Figures 6B,D**). Therefore, we confirmed that damages by needle insertion itself were absent in the sciatic nerves (**Figures 6A–D**). Furthermore, we confirmed that this demyelination model did not cause axonal damages because many neurofilament positive axons in LPC (+) group were detected (**Figure 6B**). Seven days after the injection to the sciatic nerve, saline or MeCbl was systemically administered with an osmotic pump. In the LPC (-) groups with or without MeCbl, the numbers of MBP positive axons are similar (**Figures 6E–H,M**) and same as that in normal sciatic nerve (**Figure 6A**) 7 days after systemic administration (14 days after the injection to the sciatic nerve). These results indicate that MeCbl did not affect the myelin of the normal sciatic nerve. The number of MBP positive axons decreased to 7% compared with the normal nerve 7 days after the LPC injection (**Figures 6B,D**), but it spontaneously increased to 41% 14 days after the LPC injection (**Figures 6I,J,M**). An administration of MeCbl for 7 days accelerated the recovery of remyelination and number of MBP positive axons was approximately twofold compared with that in LPC (+) group without MeCbl (**Figures 6I–M**). These results demonstrate that MeCbl promotes the remyelination of LPC-induced demyelination rat model.

**FIGURE 7:**
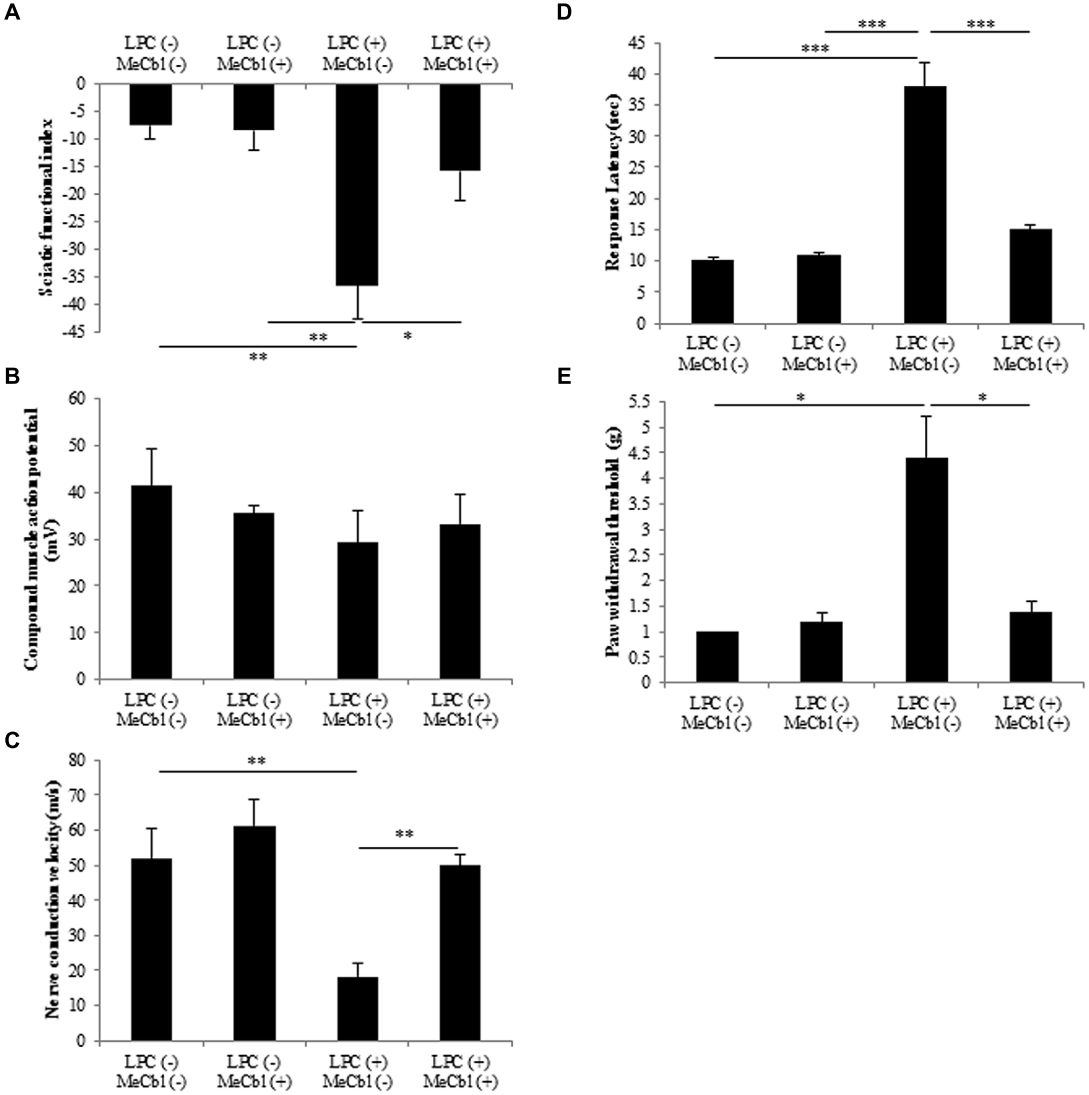
**Methylcobalamin improved the recovery of demyelinated sciatic nerve function**. To evaluate sciatic nerve motor and sensory functions, sciatic functional index **(A)**, electrophysiological studies **(B)** compound muscle action potential: **(C)** nerve conduction velocity, hot plate test **(D)** and von Frey monofilament test **(E)** were performed at 2 weeks after the injection of the drug. *p < 0.05, **p < 0.01, ***p < 0.001. Values are means ± SEM of five independent experiments.

To evaluate motor functional recovery, we administered a sciatic functional index test to the rats. At 2 weeks after the LPC injection, sciatic functional index values in the MeCbl (+) group were significantly higher than those recorded in the MeCbl (-) group (**Figure 7A**). For electrophysiological evaluation, compound muscle action potential and nerve conduction velocity were obtained by stimulating sites proximal and distal to the injection. Regarding compound muscle action potential, the amplitude was not lowered by the LPC injection and MeCbl did not affect it (**Figure 7B**). This was presumably because compound muscle action potential is affected by the number of axons and LPC does not lower the number of axons (**Figure 6B**). On the other hand, MeCbl treatment kept nerve conduction velocity at a normal level, whereas the LPC injection decreased nerve conduction velocity to a value <20 m/s (**Figure 7C**). These functional evaluations show that MeCbl promotes functional recovery in rat sciatic nerve demyelination models. The response latency on hot plate significantly increased by approximately 3.8 and 3.5-fold (**Figure 7D**) in LPC (+) MeCbl (-) group compared to those in LPC (-) MeCbl (-) and LPC (-) MeCbl (+) groups. Treatment with administration of MeCbl for LPC injection significantly improved of response latency in administration of saline for LPC injection (**Figure 7D**). The von-Frey monofilament test was employed to determine withdrawal thresholds to mechanical stimuli applied on the hind paw. MeCbl treatment for LPC injection significantly reduced compared with saline treatment for LPC injection and was improved to control level (**Figure 7E**). These results demonstrate that MeCbl promotes motor and sensory functional recovery in a rat demyelination model.

## Discussion

In this study, the *in vitro* differentiation of SC and *in vivo* remyelination were examined after MeCbl administration. MeCbl suppressed Erk1/2 activities in SCs 30 min and 1 h after the administration of the compound and promoted their differentiation in the differentiation medium but not in the growth medium. Moreover, MeCbl promoted the *in vitro* myelination of SC and accelerated remyelination after LPC induced demyelination *in vivo*.

Vitamin B plays a very critical role in the maintenance of the nervous system. For a young child, vitamin B12 deficiency brings about brain atrophy with retarded myelination (Lovblad et al., 1997). For patients after gastrectomy, absorption of vitamin B12 is impeded owing to lack of the intrinsic factor and it causes subacute combined degeneration of the spinal cord (Scalabrino et al., 1990). Niacin (vitamin B3) also plays an important role to maintain the normal function of the nervous system. 6-aminonicotinamide is a niacin antagonist and its administration causes acute damage of the gray matter in the brainstem (Penkowa et al., 2004) and the reactive astrocytes are more sensitive to 6-aminonicotinamide induced neurotoxicity than normal astrocytes (Politis, 1989). 6-aminonicotinamide also causes demyelination of the peripheral nervous system while bringing about slight effects on neurons (Friede and Bischhausen, 1978). In this study, SCs in the differentiation stage are more susceptible to the effect of MeCbl than those in the proliferation stage (**Figures 1, 4**, and **5**). Judging from these points, vitamin B may be influential especially in highly metabolic cells such as reactive astrocytes and differentiated SCs.

The Ras/Raf/Erk signaling pathway can regulate differentiation in several cell types. In SCs, sustained Ras/Raf/Erk signaling blocks the transition from immature SCs to promyelin SCs, acts as a dedifferentiation signal, and regulates myelination negatively (Harrisingh et al., 2004). Regarding the Akt signaling, its sustained activity is crucial for initiation of SCs myelination (Ogata et al., 2004). Thus, inhibiting the activity of the Erk1/2 signaling pathway and/or promoting the activity of the Akt signaling pathway is important for the *in vitro* differentiation of SC. MeCbl inhibited the activation of Erk1/2 30 min and 1 h after its addition (**Figure 2B**), while MeCbl did not promote the activity of Akt for 3 h (**Figure 2D**). Although the reason why MeCbl does not affect SC proliferation (**Figure 1**) and differentiation (**Figure 4**) in the growth medium is unknown, the temporary inactivation of Erk1/2 and the unchanged activity of Akt after the addition of MeCbl may have influenced the results. On the other hand, in the differentiation medium MeCbl promoted the expression of MBP and Acly (**Figures 4C,D**), whereas it did not affect the expression of P0 and MAG (**Figures 4A,B**). Because the expression of P0 was observed even in the growth medium and the expression of P0, MAG, and MBP reaches the plateau at 36–48 h under the differentiation medium (Leitman et al., 2011; Wu et al., 2011), it seemed to be difficult to utilize our experimental method cultured without axons as an estimation of the expression for promyelinating markers such as P0 and MAG and we also presumed that SCs culture without DRG axons for longer period such as 4 or 5 days is not appropriate for an estimation for the MBP expression. MeCbl neither affect SCs under the growth medium (proliferation in **Figure 1**; the Akt activity in **Figures 2C,D**; the expression of myelination markers in **Figure 4**) nor promote the expression of MBP at 7 days, earlier stage in the differentiation process, in cocultured DRGs/SCs (**Figures 5A–F**). Therefore, MeCbl would not be a trigger for the transition from proliferation to differentiation stage of SCs and may affect SCs only in the differentiation stage.

After peripheral nerve injury, Erk1/2-mediated signaling is important for the normal SC response (Napoli et al., 2012), and the activity of Erk1/2 is promoted in both the proximal and distal nerve stumps (Harrisingh et al., 2004). SCs can dedifferentiate and proliferate in response to nerve injury via the activation of Erk1/2 as part of a process called Wallerian degeneration. This phenomenon of SCs proliferation after peripheral nerve injury had been recognized to be prerequisite for regeneration. However, the report using mice lacking cyclin D1 revealed that SCs proliferation was not necessary for functional recovery after peripheral nerve injury (Yang et al., 2008). Furthermore, in the wild type littermates, newly generated SCs after peripheral nerve injury were culled by apoptosis (Yang et al., 2008). Because MeCbl did not influence the number (**Figure 1**) and apoptosis (**Figure 3**) of SCs under the proliferation condition, MeCbl might regulate SCs condition neither positively nor negatively in the SCs proliferation stage during Wallerian degeneration. Only in the following stage of SCs redifferentiation (remyelination), MeCbl might regulate SCs condition positively as it promoted the differentiation of SCs *in vitro* (**Figures 4** and **5**) and remyelination *in vivo* (**Figure 6**). Moreover, the upregulation of the Erk signaling pathway was observed in patients with neurofibromatosis type 1 with the loss of neurofibromin in SCs (Parrinello et al., 2008) and in leprosy patients with demyelination (Tapinos et al., 2006). Therefore, the regulation of Erk1/2 activity may be essential for the maintenance of normal peripheral nerve function and regeneration after peripheral nerve injury. MeCbl may be a promising treatment for peripheral nerve injury because it inhibited the Erk1/2 activity of SCs in the proliferation (**Figure 2**) and promoted the differentiation of SCs *in vitro* (**Figures 4** and **5**) and *in vivo* (**Figure 6**).

The peripheral nervous system consists of neurons (axons), SCs, and muscles, if axons are of motoneurons. There are some reports that MeCbl, which is an analog of vitamin B12, brings about a favorable effect on the nervous system. In *in vivo* studies, the administration of MeCbl promoted nerve regeneration in streptozotocin-diabetic rats (Sonobe et al., 1988), acrylamide neuropathy rats (Watanabe et al., 1994), gracile axonal dystrophy mutant mice (Yamazaki et al., 1994), and sciatic nerve injured rats (Okada et al., 2010). In a previous report, we demonstrated that MeCbl promotes the activities of Erk1/2 and Akt in neurons (Okada et al., 2010). Furthermore, we found that MeCbl promotes the proliferation and migration of C2C12 myoblast cells and inhibits apoptosis during the differentiation process via the Erk1/2 signaling pathway (Okamoto et al., 2014). Muscle tissue condition is very important for regeneration after peripheral nerve injury because it is the final target of motoneuron axons and its degeneration would be incurable after a prolonged denervation. In this report, we found a novel beneficial effect of MeCbl on SCs because it played important roles in regeneration after peripheral nerve injury. Previously we have already showed the promotion of myelination of the severed sciatic nerve in rats (Okada et al., 2010). During the Wallerian degeneration, injured axons regenerate first and as the next step dedifferentiated SCs go toward the redifferentiated stage in contact with regenerated axons. In the previous rat model, the regeneration of myelination seemed to be the secondary effect after the axonal regeneration. In this *in vivo* study, we therefore used an LPC-induced demyelination model to examine simply the effect of MeCbl on SCs without damages to the axons (**Figure 6B**). In this model, MeCbl promoted remyelination after demyelination (**Figure 6**). Furthermore, we analyzed the efficacy of MeCbl treatment regarding motor and sensory functional recovery in sciatic nerve demyelination rat models (**Figure 7**). LPC is a main plasma component that is synthesized after tissue injury and converted to lysophosphatidic acid by autotaxin, subsequent causing nerve demyelination and neuropathic pain via an identified mechanism (Wallace et al., 2003; Inoue et al., 2008; Nagai et al., 2010). These findings led us to conclude that MeCbl may be effective in the treatment of LPC-induced neuropathic pain. Thus, the administration of MeCbl may be one of the treatments for peripheral nerve injury, neuropathic pain, and demyelinating diseases.

## Author Contributions

SN performed all experiments. SN, MO, and KO performed analysis. SN, HT, TM, and HY interpreted the data and wrote the paper.

## Conflict of Interest Statement

The authors declare that the research was conducted in the absence of any commercial or financial relationships that could be construed as a potential conflict of interest.

## Abbreviations

Acly: ATP citrate lyase
db-cAMP: dibutyryl adenosine 3’,5’-cyclic monophosphate
DRG: dorsal root ganglion
LPC: lysophosphatidylcholine
MAG: myelin associated glycoprotein
MBP: myelin basic protein
MeCbl: methylcobalamin
NF200: neurofilament 200
P0: myelin protein zero
SCs: Schwann cells
TNF-α: tumor necrosis factor-α.
